# Guardians’ Self-Reported Fair/Poor Oral Health Is Associated with Their Young Children’s Fair/Poor Oral Health and Clinically Determined Dental Caries Experience

**DOI:** 10.3390/ijerph20010632

**Published:** 2022-12-30

**Authors:** Kaitlin E. Jones, Miguel A. Simancas-Pallares, Jeannie Ginnis, Poojan Shrestha, Kimon Divaris

**Affiliations:** 1 Division of Pediatric and Public Health, Adams School of Dentistry, University of North Carolina-Chapel Hill, Chapel Hill, NC 27599, USA; 2 Department of Epidemiology, Gillings School of Global Public Health, University of North Carolina-Chapel Hill, Chapel Hill, NC 27599, USA

**Keywords:** children, caregivers, self-reported measures, early childhood caries, epidemiology

## Abstract

In this cross-sectional, community-based study among a multi-ethnic sample of preschool-age children in North Carolina, United States, we sought to quantify the association between guardians’ self-reported oral health and their children’s oral health and determine whether race/ethnicity and education level modify these associations. We used questionnaire (n = 7852) responses about caregivers’ and their children’s oral health and clinical examination-derived (n = 6243) early childhood caries (ECC) status defined at the ICDAS ≥ 3 caries lesion detection threshold. We used multi-level mixed-effects generalized linear models to examine the associations between the guardians’ reported oral health and their children’s reported and clinically determined oral health among the entire sample and within strata of race/ethnicity, guardians’ education, and children’s dental home. The guardians’ and their children’s reported fair/poor oral health (FPOH) were 32% and 15%, respectively, whereas 54% of the children had ECC and 36% had unrestored disease. The guardians’ FPOH was strongly associated with their children’s FPOH (average marginal effect (AME) = +19 percentage points (p.p.); 95% CI = 17–21), and this association was most pronounced among Hispanics, lower-educated guardians, and children without a dental home. Similar patterns, but smaller-in-magnitude associations, were found for the guardians’ FPOH and their children’s clinically determined ECC (AME = +9 p.p.; 95% CI = 6–12) and unrestored disease (AME = +7 p.p.; 95% CI = 4–9). The study’s findings support a strong association between guardians’ and their children’s reported and clinically determined oral health and implicate ethnicity, education, and having a dental home as factors possibly modifying the magnitude of these associations.

## 1. Introduction

Early childhood caries (ECC) is the most prevalent non-communicable disease among preschool-age children and a persistent global public health problem [1,2]. The disease imposes substantial human and economic costs on children, their families, and the public health care infrastructure [3]. This burden falls disproportionately on socially and economically disadvantaged families whose children have elevated ECC risk compared to their less-disadvantaged counterparts and who may be unable to access requisite dental care [4,5]. Understanding the social and family context during the first years of life is therefore essential in efforts to identify children who may be at the highest risk for ECC.

From an etiologic standpoint, our current understanding of dental caries is that of a diet-mediated microbial dysbiosis at the tooth surface-biofilm interface resulting in a drop in local biofilm pH, progressive tooth surface demineralization, and eventual structure loss (i.e., cavitation) [6,7]. The observed associations [8,9,10,11] between parents’ and their children’s oral health, e.g., the mothers’ or fathers’ caries experience and their children’s dental caries experience, are likely to be influenced by multi-level and possibly interacting biological, behavioral, and social factors [12,13,14,15,16]. While a complete determination of all these factors is inefficient and likely unfeasible, using parents’ oral health to infer their children’s ECC propensity can offer an efficient way to identify at-risk children early, help prevent disease from developing, or seek care in a timely manner.

It stands to reason that guardians’ self-reported oral health, an arguably easily collectable report that is commonly asked in national surveys and can be easily included in clinic questionnaires or public health surveys, is a promising proxy for their children’s reported and clinically determined oral health. Several studies have examined this association among small samples of caregiver–child dyads, e.g., among 187 mothers residing in a rural, primarily Hispanic community in California [9] and among two samples of caregiver–infant dyads totaling approximately 200 children in Iowa [11]. Both studies found that sub-optimal maternal oral care (e.g., presence of untreated caries lesions and self-reported tooth loss) was associated with dental caries experience in their offspring. Despite the logical and few empirical connections made between maternal and child oral health, the magnitude of these associations in large, community-based samples and whether they are influenced by families’ sociodemographic characteristics remain unknown. To address this knowledge gap, we sought to quantify the association between guardians’ self-reported oral health and their children’s reported (i.e., subjective) and clinically assessed (i.e., objective) oral health and determine whether important socio-demographic characteristics such as race/ethnicity and education, and whether children have a dental home modify these associations.

## 2. Materials and Methods

We used questionnaire (n = 7852) and clinical examination (n = 6243) data from a multi-ethnic, community-based study of early childhood oral health conducted in North Carolina, United States, between 2016 and 2019. More information on sampling and participant recruitment protocols can be found in previous publications [17,18]. In brief, participating children were between the ages of 36 and 71 months, comprised a state-wide sample covering 86 out of 100 total counties, and were enrolled in 34 public preschool systems (Head Start programs) encompassing 260 public preschool centers. All children between the ages of 3 and 5 years who attended public preschools in the 34 sampled programs in the 86 NC counties and had a legal guardian at least 18 years old who was able to understand the study’s informational material and consent documents (provided in the English and Spanish language) were invited to participate in the study (n = 13,089). There were no important differences in key demographic characteristics between the children who received a dental examination and those who were absent from school and did not receive a dental examination at the time of the study conduct [17]. All participating children’s legal guardians provided written informed consent, and the study was approved by the UNC-Chapel Hill IRB (#14-1992).

Socio-demographic information and reported oral health data were collected via a 15-item questionnaire including NHANES-type questions asking about the guardians’ own and their children’s oral health status, i.e., ratings of excellent/very good/good/fair/poor. The guardians’ educational attainment was reported as some elementary, some high school, high school/GED diploma, some technical/college education, and college or more. Less than high school education was considered “lower education” for the purposes of this study. Race/ethnicity was categorized as non-Hispanic Black (African American), non-Hispanic White (Caucasian), Hispanic/Latino, and other or more than one race. Presence of a dental home [19] was defined as children reportedly having visited a dentist for routine care (i.e., “cleaning and check-up”) and not only because of a problem. The 15 questions alongside information about missing responses were presented in detail in a previous report [17].

The children’s clinical examinations were done at their preschools by trained and calibrated clinical examiners using ICDAS criteria as previously reported [20]. Clinical examinations were completed in approximately 80% of enrolled participants, with a detailed flowchart of inclusion and exclusion criteria reported in a previous cohort profile publication [17]. For the purposes of this study, ECC was defined as one or more primary tooth surfaces with caries experience, i.e., decayed, missing due to caries, or filled primary tooth surfaces (dmfs) index ≥ 1, wherein the ‘d’ component was assessed at the International Caries Detection and Assessment System (ICDAS) ≥ 3 caries lesion detection threshold. Unrestored disease was defined as one or more primary tooth surfaces with unrestored caries lesions (i.e., ds ≥ 1).

We used descriptive methods for initial data presentation and then relied on multivariable modeling based on multi-level mixed-effects generalized linear models with a logit link to examine the associations of the guardians’ reported oral health with their children’s reported and clinically determined oral health (i.e., ECC case status and untreated disease). There was no evidence of collinearity between the oral health outcomes of interest (i.e., Pearson’s correlation coefficients were 0.45 between the children’s and their guardians’ reported oral health and 0.38 between the guardians’ oral health and their children’s dmfs index). For analytical purposes, the guardians’ and their children’s reported oral health statuses were dichotomized as fair/poor versus excellent/very good/good consistent with previous public health investigations among adults [21] and children [22]. Because odds ratios tend to overestimate the magnitude of associations when the modeled outcomes are common, we used marginal effects estimation [i.e., average marginal effects, AME, and 95% confidence intervals (CI)] to quantify associations on a prevalence difference scale, i.e., differences in adjusted prevalence expressed in percentage points (p.p.) [23]. To determine whether Hispanic ethnicity, guardians’ education, and children having a dental home modified the observed associations, we conducted analyses stratified by these factors and qualitatively compared the stratum-specific AME estimates. We used Stata 17.0 (StataCorp LLC, College Station, TX, USA) for all analyses.

## 3. Results

Descriptive information of the children and guardians who were included in the analytical sample is presented in Table 1. Of note, 2.6% of the responding guardians had missing information for the self-reported oral health status question, and thus were excluded from this study, leaving 7852 participants with non-missing responses. African Americans (non-Hispanic Blacks) were the most represented in the study sample (48%) whereas one-fifth were of Hispanic ethnicity. Almost one out of five of the guardians had completed less than high school education. The children’s mean age was 52 months and the vast majority reported having a dental home.

The guardians’ and children’s reported fair/poor oral health (FPOH) were 32% and 15%, respectively, with 10% and 22% reporting excellent adult or child oral health (Table 2). More than half of the children (54%) were found to be ECC cases and a little over a third (36%) had unrestored disease. The low-educated guardians were more likely than the higher-educated ones to have FPOH (40% versus 30%, *p* < 0.00005) and in turn, the children of the guardians with FPOH were less likely to have a dental home compared to the guardians with better oral health (79% versus 86%, *p* < 0.00005). There were no differences in the guardians’ reports of fair/poor oral health associated with ethnicity.

Multivariable models adjusting for children’s age, sex, race/ethnicity, reported dental home, and guardians’ education level revealed strong associations between guardians’ FPOH and their children’s FPOH (average marginal effect (AME) = +19 percentage points (p.p.); 95% CI = 17, 21) (Table 3). Similar associations, but smaller in absolute magnitude, were found for clinically determined measures of oral health. Specifically, the guardians’ reports of their own FPOH were associated with an adjusted average 9 percentage points (95% CI = 6, 12) higher prevalence of ECC among their children and 7 percentage points (95% CI = 4, 9) higher prevalence of unrestored disease. These estimates were comparable but lower compared to the crude, unadjusted prevalence differences (e.g., the children’s FPOH, 32% versus 7%, a 25-percentage-point crude difference).

Stratified analyses by ethnicity (Hispanic versus non-Hispanic), guardians’ education (less than high school versus high school or more) and their children’s reported dental home revealed important differences in the magnitude of the studied associations (Table 4). The association between the guardians’ and their children’s reported FPOH was approximately 10 percentage points stronger among those of Hispanic ethnicity, less than high school education, and children without a dental home. It is important to note that these associations were independent, i.e., adjusted for the influence of each other factor in multivariable models. Similar but smaller-in-magnitude differences were found in the associations of the guardians’ reported FPOH with ECC and unrestored disease between strata of Hispanic ethnicity and dental home. For example, the guardians’ FPOH was associated with 10 p.p. higher prevalence of unrestored disease among Hispanics and those without a dental home compared to 6 p.p. increase among non-Hispanics and those with a dental home. On the other hand, a noteworthy association between the guardians’ FPOH and ECC was only evident among those with at least high school education (+10 p.p.; 95% CI = 7, 13) compared to only +5 p.p. (95% CI = −2, 12) among those with fewer years of education.

## 4. Discussion

In this cross-sectional study among a large, multi-ethnic community-based sample of guardians and their preschool-age children in North Carolina, United States, we found a strong association between the guardians’ reports of their own fair/poor oral health and sub-optimal oral health among their young children. These associations were evident in terms of both subjective child oral health as well as clinically determined ECC and unrestored disease. The magnitude of these associations was considerable, approximately 20 percentage points for subjective reports and 7–9 percentage points for clinical measures. More importantly, with few exceptions, these estimates of association were larger among vulnerable population sub-groups, namely, Hispanics, caregivers with less than high school education, and children without dental homes. Evidence from this study adds to the knowledge base linking guardians’ and children’s oral health and implicates important social and demographic characteristics as factors possibly amplifying these associations.

The identified associations are in general agreement with previous reports in the literature. Talekar and colleagues [24] used US-representative data and found that 11% of young children’s parents rated their children’s oral health as fair/poor, an estimate comparable to what was found (15%) in the present study. Dye and colleagues found that the children of mothers with untreated dental caries were three times as likely to have untreated dental caries themselves [25]. Several other investigations carried out in diverse settings relied on clinical or guardian-reported data and consistently and positively associated parents’ oral health with that of their children’s [26,27,28,29]. From a practical, clinical, and public health standpoint, modifiable oral health behaviors (e.g., diet and oral hygiene) [30], guardians’ oral health literacy [15], and dental neglect [31] are logical targets for interventions seeking to break the intergenerational cycle of poor oral health. Of course, upstream, social factors [5,32] are the major disparity-generating forces and the strongest influences on both guardians’ and children’s oral health.

Despite the well-documented clinical associations in the literature, the strong association between the guardians’ reports and their children’s subjective oral health in this study is not surprising because both reports were provided by the same adult respondent. Similar, albeit smaller-in-magnitude associations were found for clinical measures of children’s oral health. On the other hand, the guardians’ perceptions of their young children’s oral health are valuable as measures themselves as we have shown in a recent report emanating from the same cohort [33] and another community-based sample of child–caregiver dyads [34]. Although recommendations for early preventive dental visits are abundant and widely disseminated, most dental care-seeking among preschool-age children tends to be problem-initiated [29], and in this regard, the guardians’ perceptions of their children’s oral health may be more influential than what is assessed in detailed clinical examination in terms of determining dental care-seeking and perhaps oral health-related behaviors.

This study is limited by its reliance on a single questionnaire item for the assessment of the guardians’ oral health status versus the conduct of a clinical examination. On the other hand, the self-reported questionnaire item is the one included in the U.S. national health and nutrition examination survey (NHANES) [35] and provides an efficient means to screen for individuals with oral health problems (i.e., fair/poor reported oral health). Moreover, conducting oral/dental examinations among all young children’s guardians would not be feasible from a public health system or program standpoint, and valid screening-type information is most desirable. Additionally, while the study was conducted in a large, community-based (i.e., not clinic-derived) sample of preschool-age children in one US state, it may not be representative of all 3–5-year-old children in North Carolina or other states. This is because ZOE 2.0 study participants were recruited from public preschools where enrollment criteria (i.e., income and additional social factors) are typically met by low-income and minority families. While the study population is not inclusive of middle- and upper-income groups, it is arguably the low-income and most vulnerable segment of the population that is in most need of ECC surveillance programs and screening activities.

Acknowledging the study’s limitations, the findings may be applicable to public health and clinical settings and population-level surveillance efforts. Relatively brief screening questionnaires could be developed in various languages to gather the information used in this study (e.g., the guardians’ self-reported oral health status, education, and ethnicity) and be used to make inferences or screening-type data-driven predictions for children’s ECC propensity. Machine learning-based applications are now being developed to aid in children’s oral health assessments using information from both parents and children [36]. In a recent example, an automated machine learning-based ECC screener was developed using only two inputs, i.e., caregiver reports of their children’s oral health status and the children’s age in months [37]. It is logical to expect that gains in the performance and utility of such applications will be gained with the addition of high-quality information reflecting family, social, demographic, and other contextual characteristics. The current study provides evidence for the relevance of guardians’ reports of their own oral health for better understanding ECC propensity and illuminates the influence of social and demographic factors on the association between their and their young children’s oral health.

## 5. Conclusions

The study’s findings provide support for a strong association between guardians’ and their children’s reported and clinically determined oral health in the community and implicate ethnicity, education and children’s dental homes as factors possibly modifying the magnitude of these associations.

## Figures and Tables

**Table 1:** Socio-demographic information of ZOE 2.0 study participants included in the analytical sample.

	*n* (%)
Entire sample *	7852 (100)
**Child’s age**	
3 years	2525 (32)
4 years	4108 (52)
5 years	1219 (16)
months, mean (SD)	52 (7)
**Sex ^†^**	
boy	3894 (50)
girl	3957 (50)
**Race/ethnicity**	
non-Hispanic Black	3765 (48)
non-Hispanic White	1411 (18)
Hispanic	1549 (20)
more than one/other	1127 (14)
**Dental home**	
yes	6433 (84)
no	1264 (16)
**Guardians’ educational attainment**	
some elementary	350 (5)
some high school	1090 (14)
high school/GED diploma	2918 (38)
some technical/college education	2236 (29)
college or more	1120 (15)

* Among participants with non-missing information on the guardians’ reported oral health status. ^†^ The questionnaire asked whether the participating child was a boy or a girl; subsequently, biological sex was determined via genotyping.

**Table 2:** Guardians’ and children’s reported oral health status and the children’s clinically determined oral health status (early childhood caries, ECC).

	*n* (%)
**Guardians’ reported oral health status**	
excellent	765 (10)
very good	1727 (22)
good	2861 (36)
fair	1859 (24)
poor	640 (8)
**Children’s reported oral health status**	
excellent	1748 (22)
very good	2463 (32)
good	2393 (31)
fair	1028 (13)
poor	168 (2)
Children’s * clinically determined oral health status ^†^	
no ECC	2876 (46)
ECC	3367 (54)
Children’s * clinically determined unrestored disease ^‡^	
no unrestored disease	4002 (64)
unrestored disease	2241 (36)

* Clinical examinations were conducted among 6404 (~80%) of all enrolled children. **^†^** Defined as ≥ 1 primary tooth surfaces with caries experience, i.e., dmfs > 0 wherein the ‘d’ component was assessed at the ICDAS ≥ 3 caries lesion detection threshold. **^‡^** defined ≥ 1 primary tooth surfaces with an unrestored caries lesion, i.e., ds > 0 wherein the ‘d’ component was assessed at the ICDAS ≥ 3 caries lesion detection threshold.

**Table 3:** Associations between guardian-reported oral health (fair/poor versus excellent/very good/good) and their children’s subjective oral health (fair/poor versus excellent/very good/good) and clinically determined ECC estimated as model-adjusted * average marginal effects and expressed in percentage points (p.p.) and associated 95% confidence intervals (CI).

	Guardians’ Reported Oral Health Status		Estimates of Association with Children’s Oral Health
	Excellent, Very Good, or Good	Fair or Poor	Adjusted Average Marginal Effect
	*n (column %)*	*n (column %)*	Percentage Points (p.p.) and 95% Confidence Interval
entire sample, n (row %)	5323 (68)	2477 (32)	
**Children’s subjective oral health**			
excellent/very good/good oral health	4931 (93)	1673 (68)	referent
fair/poor reported oral health	392 (7)	804 (32)	+19 p.p. (+17 p.p., +21 p.p.)
**Clinically determined oral health**			
not ECC case (dmfs = 0)	2102 (50)	774 (39)	referent
ECC case (dmfs ≥ 1)	2139 (50)	1228 (61)	+9 p.p. (+6 p.p., +12 p.p.)
**Unrestored disease**			
no unrestored disease (ds = 0)	2841 (67)	1161 (58)	referent
unrestored disease (ds ≥ 1)	1400 (33)	841 (42)	+7 p.p. (+4 p.p., +9 p.p.)

* Obtained via a multi-level mixed-effects generalized linear model adjusting for the children’s age, race/ethnicity, reported dental home, and the guardians’ education level. The reported average marginal effects are adjusted absolute differences on the prevalence scale (i.e., percentage points).

**Table 4:** Differences in the association between the guardians’ reports of their own fair/poor oral health with their children’s subjective reports of fair/poor oral health and clinically determined early childhood caries (ECC). Estimates of association are presented as model-adjusted * average marginal effects (AME) and expressed in percentage points (p.p.) and associated 95% confidence intervals (CI).

	Guardians’ Ethnicity		Guardians’ Education		Reported Dental Home	
	Hispanic	Non-Hispanic	<High School	≥High School	Yes	No
	AME, p.p. (95% CI)	AME, p.p. (95% CI)	AME, p.p. (95% CI)	AME, p.p. (95% CI)	AME, p.p. (95% CI)	AME, p.p. (95% CI)
Children’s oral health						
fair or poor subjective oral health	27 (23, 31)	17 (15, 19)	27 (24, 31)	18 (15, 20)	17 (15, 20)	28 (25, 32)
clinically determined ECC	11 (5, 17)	8 (5, 11)	5 (−2, 12)	10 (7, 13)	8 (5, 12)	11 (6, 15)
unrestored disease	10 (4, 16)	6 (4, 9)	6 (0, 13)	8 (5, 10)	6 (4, 9)	10 (3, 16)

* Obtained via a multi-level mixed-effects generalized linear model including terms for children’s age, race/ethnicity, reported dental home, and their guardians’ education level. The reported average marginal effects are adjusted absolute differences on the prevalence scale (i.e., percentage points).

## Data Availability

The study’s data are available via the Carolina Digital Repository under study name “ZOE 2.0: A community-based, epidemiologic study of early childhood oral health” and are accessible via: https://cdr.lib.unc.edu/concern/data_sets/kk91fv385 (accessed on 24 October 2022).
